# Treating a patient with ADP porphyria through suppression of both hepatic and erythroid ALA production

**DOI:** 10.1186/s13023-026-04470-8

**Published:** 2026-07-13

**Authors:** Niels C. Veldhoen, Nicole C. Peltenburg, Eduard J. van Beers, Edith C. H. Friesema, Janneke G. Langendonk

**Affiliations:** 1 Porphyria Expertcenter Rotterdam, Center for Lysosomal and Metabolic Disease, Department of Internal Medicine, Erasmus MC, University Medical Center, Rotterdam, The Netherlands; 2 Department of Hematology, UMC Utrecht, University Medical Center, Utrecht, The Netherlands

## Abstract

δ-Aminolevulinic acid dehydratase deficient porphyria (ADP) is an exceedingly rare form of acute porphyria, with only fifteen published cases worldwide. This disorder has historically been considered an hepatic porphyria, as it was thought the overproduction of the toxic metabolite δ-Aminolevulinic acid (ALA) solely occurred in the liver. This case report demonstrates that treatments focused on reducing the hepatic ALA production had limited effect on this patient’s symptoms. An earlier published hypothesis that patients with ADP also produce substantial levels of bone marrow derived ALA, was retested. We describe a Dutch patient in his early 60s, who has suffered from ADP since birth. His medical history mentions acute neurovisceral attacks, contractures of hand and feet, severe polyneuropathy, developmental delay, and renal insufficiency (eGFR 30–40 ml/min). For over a decade he was treated with weekly heme prophylaxis to reduce the frequency of neurovisceral attacks. Despite continuation, his chronic neurological symptoms aggravated and, in an attempt to suppress erythroid ALA production, weekly erythrocyte transfusions combined with hydroxyurea was started. The patient’s symptoms improved and stabilized during this treatment. Oral chelation therapy was started to limit secondary iron overload caused by both heme and erythrocyte infusions. Years later we could trial givosiran, an ALAS1-siRNA to suppress the hepatic ALA production. After starting givosiran the heme infusions could be stopped without an increase in symptoms, but discontinuation of erythrocyte transfusions led to new onset neurovisceral attacks. His current schedule consists of continued givosiran and erythrocyte transfusions. This case report illustrates that ADP can be treated successfully with both hepatic and erythroid suppression. It also illustrates the challenges of treating a patient with a rare, complex and poorly understood disease, and the difficulty to balance the complications, side effects and the benefits of treatments.

## Introduction

Aminolevulinic acid dehydratase (ALAD) deficiency porphyria (ADP) is the rarest and least understood form of porphyria, with only 15 confirmed cases reported worldwide [1–16]. ADP is an autosomal recessive disorder caused by pathogenic variants in the ALAD gene, which encodes the ALAD enzyme responsible for the second step in the heme biosynthesis pathway. A deficiency in this enzyme leads to elevated levels of delta-aminolevulinic acid (ALA). Severe accumulation of ALA can trigger neurovisceral attacks, typically presenting as abdominal pain and both sensory and motor neuropathy; symptoms similar to those seen in other acute porphyrias [17]. In addition to these acute neurovisceral symptoms, most patients with ADP have been reported to exhibit mild intellectual disability, contractures of the hands and feet, renal insufficiency, and hypertension [1–16].

These neurovisceral symptoms have led to the classification of ADP as an acute hepatic porphyria, however the effectiveness of treatments directed to reduce hepatic heme production are not effective as they are in other acute hepatic porphyrias [1–17]. The most commonly used treatment for acute attacks is heme infusions, as heme can suppress heme synthesis in the liver via a direct negative feedback mechanism. In severe or refractory cases with acute intermittent porphyria (AIP), liver transplantation has been performed successfully to stop acute porphyric attacks [18–20]. A more recent successful therapeutic option is givosiran, a liver-specific small interfering RNA (siRNA) that inhibits ALAS1, the first and rate-limiting enzyme in hepatic heme synthesis.

These available treatment options are not as successful in patients with ADP compared to AIP. Intravenous heme can reduce the frequency of attacks in patients with ADP, but its efficacy appears to be much lower [1–16]. One ADP patient underwent liver transplantation, which did not lead to a significant reduction in neurovisceral symptoms [4]. Furthermore, givosiran showed only limited therapeutic benefit in a recently reported ADP case [3]. In addition to these cases where treatments targeting hepatic heme synthesis had limited success, another report described a patient who developed ADP-like symptoms following the onset of polycythemia vera [21]. This patient was diagnosed with a single germline pathogenic variant in the ALAD gene, with a second variant presumed to be present in the malignant cells. On top of this, the most recent ADP publication reports a patient with ADP with somatic mosaicism, likely restricted to the hematopoietic lineage [16]. These observations have led to the hypothesis that the overproduction of ALA also occurs in erythroid cells, and subsequently plays a role in the pathophysiology of ADP, parallel to hepatic ALA production.

In accordance with this hypothesis, a treatment strategy aimed at suppressing both hepatic and erythroid ALA production was initiated in our Dutch patient. The efficacy during the first year of this treatment has been reported in 2019 [1]. The current report is an update with new strategies and six more years of follow-up, describing the optimal dosing of erythroid treatment and the effects of adding givosiran to the treatment schedule. Secondarily, we describe the complexity and long term effects of these treatments. The aim of this paper is to contribute to a better understanding of the pathophysiology of ADP and share the optimization of treatment strategies.

## Case report

**Fig. 1:**
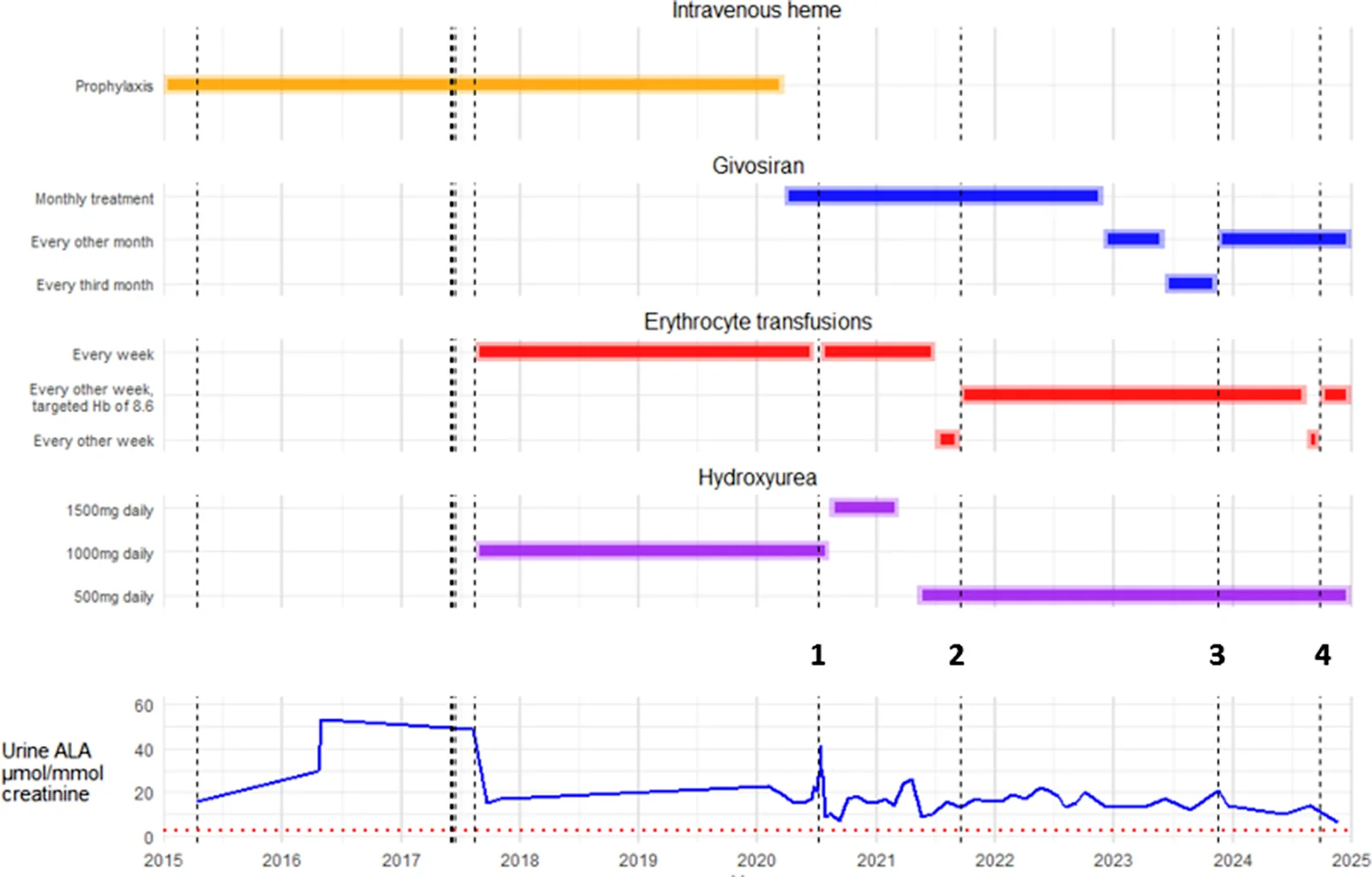
Clinical outcome of several treatment modalities in a patient with ADP. Presented treatments were intravenous heme, erythrocyte transfusions, hydroxyurea, and givosiran. The acute porphyric attacks are presented with the vertical dashed lines, and urinary ALA levels are reported in µmol/mmol creatinine. The acute porphyric attacks (admissions to the hospital due to clear neurovisceral symptoms) that occurred after the start of the erythrocyte transfusions are numbers 1–4. Attacks 1, 2 and 4 occurred shortly after a reduction in the frequency of erythrocyte transfusions, attack 3 occurred after increasing the interval between givosiran administrations

This is an update of the case report of a patient with ADP born in the sixties. His medical history was previously reported in 2019 [1]. The purpose of this update is to show the disease course following the advent of givosiran treatment combined with long term erythroid heme synthesis suppression. The details of the attempted treatment strategies are visualized in Fig. 1. The patient and his legal guardians gave written and oral consent to this publication.

After an uncomplicated birth, the patient developed tetraplegia and respiratory insufficiency on day 2 of his life, which spontaneously recovered. During childhood there were several more episodes of neurological deficits, with again spontaneous but often incomplete recovery. He developed bilateral ankle contractures, hearing disability, a mild cognitive impairment and was diagnosed with autism. Since reaching adulthood he lives independently with daily assistance combined with practical, administrative and financial support. In 2007, the patient, then aged mid-40s, presented with abdominal pain, nausea, vomiting and progressive asymmetric weakness in all extremities. His medical history mentioned unexplained progressive renal insufficiency (~ 50 mL/min). These observations eventually led to the diagnosis of ADP based on elevated urinary ALA with normal PBG, while lead intoxication, AIP and tyrosinemia were ruled out. The diagnosis was confirmed with genetic analysis, which determined the patient is compound heterozygous for two splicing variants in the *ALAD* gene (NM_000031.6:c.114–11 C > T and NM_000031.6:c.164 + 2T > A).

In the following years the patient suffered from multiple acute porphyric attacks treated with intravenous heme on demand (Hemine or heme arginate, Normosang^®^, Recordati Rare Diseases UK Ltd), and later prophylactic heme on a weekly schedule. This was initially effective in reducing his attack frequency. Years of weekly prophylactic heme was complicated by iron overload, and treatment with phlebotomies and iron chelation therapy (deferasirox) was started. In 2017 he was admitted trice within two weeks for attacks of abdominal pain, motor dysfunction, and increased neuropathic pain. In this period, therapy resistant hypertension also worsened. These problems led to a need for alternative treatment options.

**Fig. 2:**
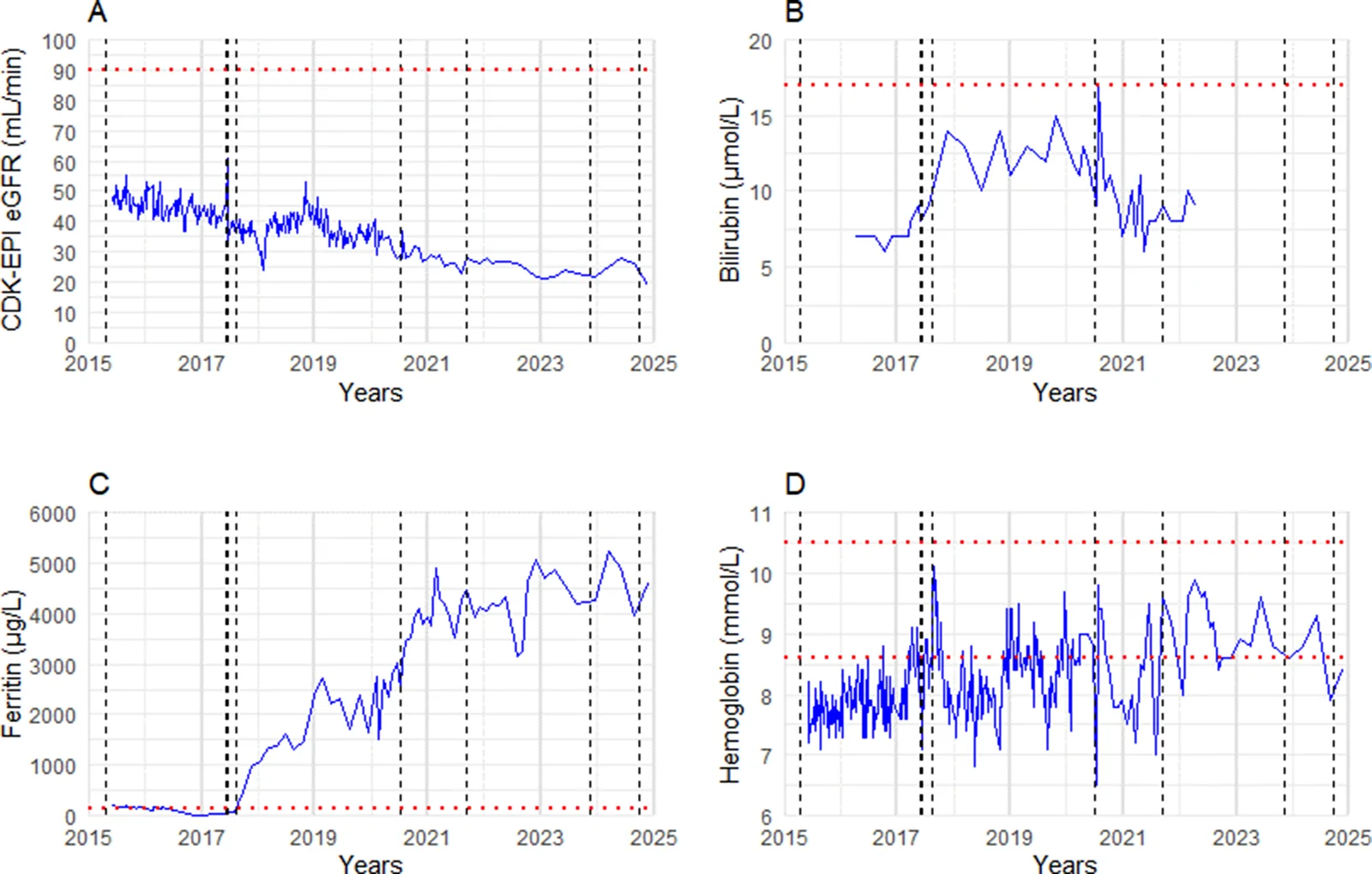
**A**: The renal function over the observational period of the reported patient with ADP, presented as estimated or eGFR, based on plasma creatinine, in mL/min. **B**: Bilirubin levels in µmol/L. **C**: Ferritin levels in µg/L. **D**: Hemoglobin levels in mmol/L. The hemoglobin levels at the start of the transfusions in august 2017 was 8.4 mmol/L. The dashed vertical lines are the timepoints when the patient presented with an attack. The dashed horizontal lines are normal reference values

Because of the limited effect of the treatments and previously mentioned ADP cases [4, 21], the hypothesis was formed that suppressing erythroid heme synthesis could reduce the attack frequency in ADP. Based on this hypothesis the phlebotomies were stopped, erythrocyte transfusions (1 packed cell every two weeks) and hydroxyurea (daily 1000 mg, Hydrea^®^) were started while heme infusions were continued weekly. Renal function (eGFR), bilirubin, ferritin and hemoglobin were continuously monitored with every transfusion (Fig. 2). Immediately afterwards the attacks ceased and his neuropathic symptoms improved.

This treatment regimen was relatively successful; he did not suffer from acute porphyric attacks, and there was no indication for a hospital admission for two years. However, the treatment was complicated by progression of the iron overload, with ferritin levels reaching over 2500 µg/L (Fig. 2C). The availability of givosiran (Givlaari, Alnylam Ltd.) in 2020 changed his treatment options. Heme was stopped immediately following the initiation of givosiran treatment, but weekly blood transfusions (now administering 2 packed cells per week) and hydroxyurea continued. This regiment with givosiran instead of heme resulted in an evident clinical improvement: he suffered less from nausea, gained weight, and daily morning vomiting diminished to sporadically, leading to an increase in BMI as well. Most notably, the substitution of intravenous heme for givosiran did not lead to an acute porphyric attack.

After four months of givosiran, an attempt was made to discontinue the blood transfusions while continuing givosiran. Ten days after discontinuation the patient was admitted with an acute porphyric attack (attack no. 1 in Fig. 1), presenting with abdominal pain, nausea, vomiting, tingling sensations in his perineum and dorsal upper legs; symptoms he recognized from previous attacks. The patient was treated with heme infusions on three consecutively days, which reduced the complaint of tingling sensations. The abdominal pain, nausea and vomiting persisted for a week. Weekly erythrocyte transfusions were restarted, and the patient made a full recovery after another week.

A year after the last attack, following concerning increases in iron accumulation (ferritin 3521 µg/L, transferrin saturation 116%, with no signs of iron accumulation in the heart, liver and kidneys visible on T2*MRI), another attempt was made to reduce the number of blood transfusions. The frequency of blood transfusions was reduced from two units every week to one unit every two weeks. One month later the patient presented once again with identical symptoms of an acute porphyric attack, which were resolved after additional blood transfusions (attack no. 2 in Fig. 1).

The transfusion schedule was adjusted to biweekly administration, with one or two units transfused according to hemoglobin level; two units were given when levels were below 8.6 mmol per liter, otherwise only one unit was administered.

The final attempt to reduce the frequency of the erythrocyte transfusion was done more than 3 years after the previous attempt, at the end of 2024. The frequency was again changed to one unit every two weeks, but 6 weeks after this change he presented again with an attack (attack no. 4 in Fig. 1). The symptoms were managed after three packed cells were administered, and his previous transfusion schedule was resumed.

Another attack was triggered by prolonging the interval of givosiran dosing (attack no. 3 in Fig. 1), while the erythroid transfusion schedule remained unaltered. This change in the treatment schedule of givosiran was prompted by a slow, but progressive decline in renal function. The renal function had been stable during the first year of givosiran (CKD-EPI eGFR 28–35 mL/min), thereafter it slowly reduced to 20–25 mL/min. This urged us to prolong the givosiran dosing interval from monthly to every 8 weeks, and later every 12 weeks. After a dosing interval of 12 weeks, the patient again presented with symptoms of an acute porphyric attack (attack no. 3 in Fig. 1): mild abdominal pain and cramps in the upper legs. The givosiran treatment interval was shortened to every 6 weeks. His renal function neither improved nor reduced during these changes. His eGFR remained 20–25 mL/min until the end of the observation window (Fig. 2A). The estimated GFR based in Cystatin C was similar. Worsening renal function necessitated a hydroxyurea dose reduction, to reduce the risk of hydroxyurea induced nephrotoxicity and reduce hematological toxicity. This dose adjustment had no clinical impact.

**Fig. 3:**
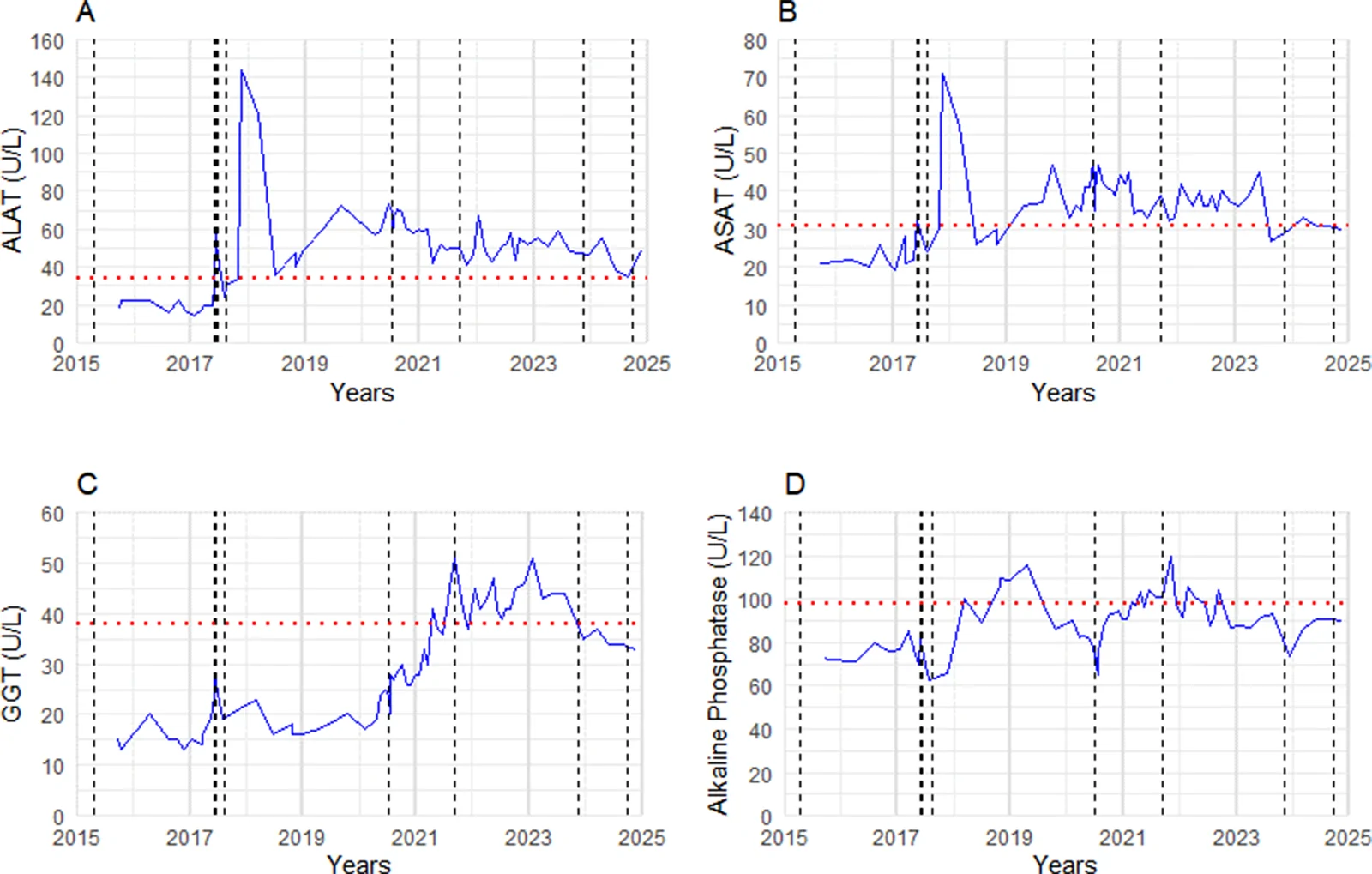
Liver enzymes over time. **A**: Alanine transaminase (ALAT) in U/L. **B**: Aspartate transaminase (ASAT) in U/L. **C**: Gamma-glutamyl transferase (GGT) in U/L. **D**: Alkaline phosphatase in U/L. The dashed vertical lines are the timepoints when the patient presented with an attack. The dashed horizontal lines are normal reference values

One of the side effects of givosiran is an increased homocysteine level in most AIP patients [22, 23]. The highest homocysteine level in this ADP patient was 24.0 µmol/L (upper limit of normal < 15 µmol/L). A baseline homocysteine level is unavailable, as this side effect was unknown when this patient started givosiran. In the last few years, he was also diagnosed with type 2 diabetes mellitus, osteoporosis and idiopathic interstitial lung disease (deemed secondary of years of daily vomiting). Liver enzymes were routinely measured before each givosiran administration and remained normal (Fig. 3).

## Discussion

This presented case of a patient with δ -Aminolevulinic acid dehydratase deficient porphyria (ADP) describes the clinical effects of several serial medical interventions. Our first conclusion is that givosiran is an adequate substitute for prophylactic heme infusions, seemingly even improving the clinical symptoms of the patient. The second conclusion is that erythrocyte transfusions are pivotal to preventing acute porphyric attacks, and both erythrocyte transfusions and givosiran are necessary to maintain the improved stable situation.

These conclusions are based on the fact that attempts to stop or reduce the frequency of the transfusions led to incident attacks within weeks. The hypothesis that the pathophysiology of ADP depends on both hepatic and erythroid ALA overproduction is therefore clinically confirmed. It should be noted that suppression of only hepatic heme production never led to a complete absence of symptoms for this patient with ADP. However, prolonging the dosing intervals of givosiran treatment was a trigger for another acute porphyric attack, confirming the involvement of both heme producing sites.

These findings, which show a limited impact from merely decreasing hepatic heme synthesis, align with previously documented cases of ADP [1–16]. Graff et al. demonstrated the limited effect of both prophylactic heme administrations and givosiran in a 32 year old patient with ADP [3], which is in stark contrast to the notable effectiveness of givosiran in patients with acute intermittent porphyria [24]. Combining these observations with an ADP case where a liver transplant did not alleviate symptoms [4] and a case where the patient acquired ADP after developing polycythemia vera [5, 6] further support the hypothesis that the erythroid heme production is involved in the pathophysiology of ADP. However, while this case offers strong evidence for this hypothesis, definitive confirmation of this hypothesis would only be possible through basic science approaches. Furthermore, it should be noted that the severity of the clinical presentation, the genetic variant of the ALAD gene and ALAD enzyme activity varies between all reported ADP patients [1, 16]. Therefore, it remains uncertain if other ADP cases would see a similar reduction of symptoms after suppressing both hepatic and erythroid heme synthesis.

This case report also describes the pitfalls and challenges for a patient with ADP, and presents an overview of the current optimal treatment schedule which prevents attack in this case. Furthermore, this case demonstrates that co-morbidities like iron overload and renal insufficiency are a major concern. We advocate to monitor patients with ADP on these complications, and act upon abnormal results. Regular blood transfusions and heme prophylaxis have led to iron accumulation, which could lead to organ damage. With the advent of givosiran we were able to stop heme, which is a reduction in weekly secondary iron admission. The combination of iron overload and renal failure is complex, as givosiran and hydroxyurea can compromise renal function, while iron chelation therapy cannot be dosed to its maximum potential with the renal dysfunction. Optimizing treatment and finding a balance between the positive and negative effects of the treatments is a challenge, and close monitoring during these therapies and adjusting the frequency and dose of all therapies is crucial.

## Conclusion

This case report illustrates the outcomes of different treatment strategies attempted in this ADP case, with the aim to find the optimal treatment for this ADP patient. Our findings indicate that ADP is not just an hepatic porphyria, demonstrated by the efficacy of the erythroid heme synthesis suppression on frequency of acute porphyric attacks. This report also demonstrates the challenge of treating a patient with this rare, complex and poorly understood disease, as it is difficult to balance the complications of his condition with the benefits and side effects of the different treatments.

## Data Availability

To protect the privacy of the patient we cannot make the data publicly available. We will make the data available upon reasonable request to the corresponding author.
